# Nebulized fentanyl does not improve exercise capacity or dyspnoea in fibrosing interstitial lung disease

**DOI:** 10.1113/EP092123

**Published:** 2024-10-12

**Authors:** Charlotte Chen, John Kolbe, Julian F. R. Paton, James P. Fisher

**Affiliations:** ^1^ Manaaki Manawa – The Centre for Heart Research, Department of Physiology, Faculty of Medical & Health Sciences University of Auckland Auckland New Zealand; ^2^ Faculty of Medical & Health Sciences, Department of Medicine University of Auckland Auckland New Zealand

**Keywords:** afferents, dyspnoea, exercise

## Abstract

Exercise intolerance and exertional dyspnoea are hallmarks of fibrosing interstitial lung disease (FILD) and are associated with worse prognosis and quality of life. Activation of pulmonary vagal afferents influences the ventilatory pattern and contributes to the sensation of dyspnoea. We tested the hypothesis that nebulized fentanyl, which might attenuate aberrant pulmonary afferent activity in FILD, reduces ventilation and dyspnoea while extending exercise endurance time (EET). In this randomized, single‐blind, placebo‐controlled study, eight FILD patients (two males, 71 ± 6 years of age) performed incremental cardiopulmonary cycle exercise tests following nebulization of either fentanyl citrate (100 µg) or 0.9% saline. Previous work indicated that this dose was unlikely to produce central effects. Comparisons between treatment conditions at rest were undertaken using Student's paired *t*‐test, and exercise data were evaluated with two‐way ANOVA with repeated measures. Dyspnoea was assessed using the Borg dyspnoea scale. Resting respiratory variables were not different following treatment with fentanyl and saline; however, resting heart rate was lower following fentanyl (*P* = 0.002) and remained lower throughout exercise compared with placebo (*P* = 0.008). Fentanyl did not increase EET (placebo 334 ± 117 s vs. fentanyl 348 ± 126 s, *P* = 0.250) although overall minute ventilation was reduced slightly (mean difference: −0.97 L/min, *P* = 0.022). There were no differences in ratings of dyspnoea intensity or unpleasantness between the conditions either at rest or at end‐exercise. Nebulized fentanyl did not improve EET or exercise dyspnoea but did decrease minute ventilation during exercise, although the extent of this reduction appears clinically insignificant. These findings suggest that nebulized fentanyl is unlikely to offer significant benefits for enhancing exercise capacity in FILD.

## Abstract (highlights)

- **What is the central question of this study?**
  Fibrotic interstitial lung disease is associated with dyspnoea and exercise intolerance. The role of bronchopulmonary vagal afferents is not completely understood. We asked whether nebulized fentanyl could attenuate sensory feedback and improve exercise capacity.
- **What is the main finding and its importance?**
  Nebulized fentanyl reduced exercise ventilation compared with placebo; however, the small magnitude of this reduction means that it is unlikely to be clinically significant. Exercise endurance time, peak ventilation and dyspnoea scores were unchanged. Nebulized fentanyl is therefore unlikely to offer significant benefits for enhancing exercise capacity in fibrotic interstitial lung disease.

## 1 INTRODUCTION

Interstitial lung disease (ILD) encompasses a diverse array of conditions that emerge as a result of inflammation and/or fibrosis of the lung parenchyma (Broaddus et al., 2016). Dyspnoea during physical activity (exertional dyspnoea) and exercise intolerance are prominent symptoms in people with ILD. Dyspnoea not only has prognostic implications for survival (Manali et al., 2008) but is also linked to diminished quality of life (Swigris et al., 2005). Current therapies of supplemental oxygen, pulmonary rehabilitation and anti‐fibrotic agents offer limited relief for exertional dyspnoea (Bajwah et al., 2013). Effective management of exertional dyspnoea in ILD requires the exploration of new pathways.

The control of breathing during exercise is multifactorial. Among the contributing mechanisms are vagal afferents in the airways and lungs, which convey visceral sensations to the CNS (Lee & Yu, 2014). People with pulmonary vagal afferent denervation exhibit slowed exercise respiratory dynamics, supporting the involvement of pulmonary vagal afferents in the modulation of ventilatory patterns (Grassi et al., 1993; Sciurba et al., 1988). Moreover, aberrant activity of pulmonary afferents appears to contribute to dyspnoea in lung disease (Guz et al., 1970; Noble et al., 1970). Opioids are used systemically for refractory dyspnoea and when nebulized directly into the lungs they have been purported to bind to opioid receptors on vagal sensory fibres to modify pulmonary afferent activity and alter respiratory sensation (Krajnik et al., 2010; Zebraski et al., 2000). Early studies with nebulized morphine in lung disease were disappointing (Barnes et al., 2016). However, investigations with fentanyl citrate, a more potent and lipophilic µ‐opioid agonist, showed promising outcomes (Coyne et al., 2002; Jensen et al., 2012). For example, in patients with chronic obstructive pulmonary disease (COPD), 50 µg of nebulized fentanyl increased cycle exercise endurance time (EET) by 25%. More importantly, the mean rate of increase in dyspnoea intensity and unpleasantness was reduced (Jensen et al., 2012).

Only one study has investigated nebulized fentanyl in ILD (Milne et al., 2020). Using fixed‐duration and constant work‐rate (WR) cycling, they found no difference in dyspnoea or pattern of ventilation at isotime. Whether nebulized fentanyl can alleviate dyspnoea and improve exercise tolerance in ILD remains unclear, because constant WR tests typically evaluate only submaximal exercise responses at a single intensity, and EET was not assessed. Incremental WR tests, in contrast, enable an assessment to be undertaken over a range of intensities including peak exercise. This is pertinent because some reflex mechanisms elicited by stimulation of pulmonary afferents require a threshold tidal volume (Coleridge & Coleridge, 2011). Incremental cardiopulmonary exercise tests (CPETs) also facilitate the identification of underlying mechanisms of exercise intolerance and provide prognostic information (American Thoracic Society & American College of Chest Physicians, 2003).

In this study, we performed a randomized controlled trial of nebulized fentanyl in patients with fibrosing ILD (FILD) using incremental CPET. FILD is a disease subtype distinguished by an elevated risk of progressive decline in lung function and premature mortality (Cottin et al., 2019). We tested the hypothesis that nebulized fentanyl can alleviate dyspnoea and improve exercise tolerance during incremental CPET.

## 2 MATERIALS AND METHODS

### 2.1 Ethics approval

This study was approved by the Healthy and Disability Ethics Committee, New Zealand (20/NTA/68) and prospectively registered in the Australian New Zealand Clinical Trials Registry (ACTRN12621000454875). Following verbal and written explanations of the study procedures, written informed consent was obtained from all participants. The study was conducted according to the *Declaration of Helsinki (2013)*.

### 2.2 Participants

Eight participants with stable FILD (no exacerbations in the preceding 6 weeks) were recruited from three respiratory clinics in Auckland, New Zealand. Eligibility criteria were physiological evidence of restriction (total lung capacity <80% predicted, forced expiratory volume in 1 s/forced vital capacity ratio >0.7) and high‐resolution CT evidence of pulmonary fibrosis (Sverzellati et al., 2015). The main exclusion criteria were >15 pack‐year smoking history, emphysema on chest CT, diagnosis of pulmonary sarcoidosis and significant comorbidities (other than FILD) that might contribute to dyspnoea and/or reduced exercise capacity. A detailed list of exclusion criteria is presented in the Supporting information (S1).

All participants attended an initial visit during which they were familiarized with the study protocols, including performance of a maximal incremental exercise test (detailed below) on an electronically braked cycle ergometer (CORTEX bike M, CORTEX Biophysik, Leipzig, Germany). Anthropometric and general health information was collected. The participants’ most recent pulmonary function test results were obtained from medical records.

### 2.3 Experimental design

The study followed a randomized, single‐blind, placebo‐controlled crossover design. Participants refrained from caffeine, alcohol and exercise for 12 h and adhered to a minimum 2 h fasting period prior to the experimental visits. Participants inhaled nebulized fentanyl citrate (100 µg; 20 µg/mL) or 5 mL normal saline (0.9% solution) placebo administered by a jet nebulizer (PARI LC Sprint, PARI, Starnberger, Germany). This dose of fentanyl is unlikely to cause systemic effects (Worsley et al., 1990). Symptoms related to opioid use were assessed (Apfelbaum et al., 2004). A venous blood sample was collected, and fentanyl levels were analysed using an enzyme‐linked immunosorbent assay (Neogen, Lexington, KY, USA). At exactly 10 min following nebulization, participants performed spirometry, then immediately underwent incremental CPET in accordance with guidelines (American Thoracic Society & American College of Chest Physicians, 2003). The protocol consisted of a 5 min resting period, followed by a 2 min warm‐up of unloaded pedalling, then 10 W/min increase in WR. Pedalling frequency was maintained at 60 rpm. Participants were given standardized verbal encouragement to exercise for as long as possible. The test ended at exhaustion or at the appearance of clinical indications for termination (American Thoracic Society & American College of Chest Physicians, 2003).

### 2.4 Cardiorespiratory measures

Participants wore an oronasal mask (Hans Rudolph Inc., Shawnee, KS, USA) attached to a spiroergometry system (Metalyzer 3B, CORTEX Biophysik). Minute ventilation (*V̇*_E_), tidal volume (*V*_T_), breathing frequency (*f*_R_), oxygen consumption (${\dot{V}}_{O_{2}}$), carbon dioxide production (${\dot{V}}_{CO_{2}}$) and respiratory exchange ratio (RER) were measured on a breath‐by‐breath basis. Heart rate (HR) and rhythm were measured by a 12‐lead ECG (custo cardio 300, custo med, Ottobrunn, Germany). Blood pressure was measured with a digital brachial artery sphygmomanometer (Tango M2 BP monitor, SunTech, Morrisville, NC, USA). Peripheral oxygen saturation ($S_{pO_{2}}$) was monitored with finger pulse oximetry (WristOx2 Model 3150, Nonin Medical, Plymouth, MN, USA).

### 2.5 Symptom measures

The intensity and unpleasantness of dyspnoea were assessed before and after nebulization, during unloaded exercise, every 2 min during loaded exercise and at the end of exercise. At each visit, it was explained to the participants that ‘intensity’ relates to ‘how intense your breathing sensation is, how much breathing sensation do you feel or how hard you are having to try to breathe’ and ‘unpleasantness’ relates to ‘how unpleasant your breathing sensation is, how bad does it make you feel’. Both symptoms were rated using Borg's 0–10 category ratio scale (Borg, 1982), with 0 corresponding to ‘not intense/unpleasant at all’ and 10 corresponding to ‘maximal intensity/unpleasantness’. In addition, the rating of leg discomfort was assessed during exercise in a similar fashion. At the end of exercise, participants were asked their reason for stopping and their rating of perceived exertion (RPE) obtained using the modified Borg RPE scale, with 0 corresponding to ‘no perceived exertion at all’ and 10 corresponding to ‘maximal perceived exertion’.

### 2.6 Data analysis

The EET was recorded from the onset of the loaded exercise to the point of exercise termination. Loaded exercise data were averaged over 15 s intervals for analysis. Identification of the estimated lactate threshold was performed using an online tool (Keir et al., 2022) which combined the ventilatory equivalents and V‐slope methods (Beaver et al., 1986). Respiratory pattern was evaluated using data from the resting period by comparing the mean and variability (coefficient of variability) of respiratory variables (*V*_T_, inspiratory time, expiratory time and inspiratory time as a percentage of tidal breath time) between treatment conditions.

### 2.7 Statistical analysis

Comparisons between placebo and fentanyl conditions were undertaken using Student's paired *t*‐test for normally distributed data and the Wilcoxon signed‐rank test for skewed data. Normality of residuals was verified by visualization. For comparisons of time series data, a relative isotime method was used. For each participant, the worst test (i.e. shortest EET) was used to identify four time points in which the two tests were segmented (25, 50, 75 and 100% of EET). The main effects of condition, time and their interaction were examined using two‐way ANOVA with repeated measures. Data are expressed as the mean ± SD unless stated otherwise. A value of *P *< 0.05 was considered statistically significant. Statistical analysis was performed using SPSS software, v.27 (IBM, Armonk, NY, USA).

## 3 RESULTS

### 3.1 Study participants

Eight participants took part in the study (two males, height 170 ± 8 cm, weight 79 ± 16 kg). The mean age was 71 ± 6 years. The most frequent FILDs were idiopathic pulmonary fibrosis (*n* = 2) and connective tissue disease‐ILD (*n* = 2). Other diagnoses included vasculitis‐associated ILD, chronic hypersensitivity pneumonitis, interstitial pneumonia with autoimmune features and pulmonary fibrosis of unclear aetiology (*n* = 1 each). There were no current smokers, and the average smoking history was 3 pack‐years. Participants had moderate restrictive disease, with mean forced vital capacity of 2.06 ± 0.68 L (65% ± 16% of predicted) and total lung capacity of 3.60 ± 0.83 L (65% ± 9% of predicted). The diffusing capacity for carbon monoxide was 3.69 ± 1.03 mmol/min/kPa (52% ± 15% of predicted). A list of comorbidities and medications is presented in the Supporting information (S2 and S3).

### 3.2 Baseline cardiorespiratory variables and spirometry

There were no differences in resting ${\dot{V}}_{O_{2}}$ and respiratory variables between placebo and fentanyl conditions (Table 1). Fentanyl did not significantly modify the breathing pattern or respiratory variability (S4). Resting HR was significantly lower after fentanyl compared with placebo (Table 1). There was no difference in spirometry after nebulization of fentanyl compared with placebo (S5).

**TABLE 1:** Cardiorespiratory responses at rest and peak exercise during an incremental cardiopulmonary exercise test.

Parameter	Rest			Peak exercise		
	Placebo	Fentanyl	*P*‐value	Placebo	Fentanyl	*P*‐value
Work rate (W)	0 ± 0	0 ± 0		57 ± 19	61 ± 20	0.102
${\dot{V}}_{O_{2}}$ (L/min)	0.29 ± 0.04	0.28 ± 0.04	0.523	0.89 ± 0.21	0.91 ± 0.21	0.667
${\dot{V}}_{O_{2}}$/kg (mL/min/kg)	3.71 ± 0.56	3.58 ± 0.81	0.553	11.64 ± 3.07	11.81 ± 2.95	0.660
RER	0.90 ± 0.05	0.90 ± 0.04	0.296	1.11 ± 0.11	1.15 ± 0.11	0.283
*V̇*_E_ (L/min)	11.71 ± 1.64	10.91 ± 2.09	0.222	40.06 ± 11.82	41.16 ± 11.90	0.571
Breathing reserve (%)	79 ± 7	80 ± 6	0.682	29 ± 24	26 ± 22	0.577
*f*_R_ (breaths/min)	20.25 ± 2.12	19.63 ± 4.87	0.588	36.43 ± 6.81	35.24 ± 5.63	0.591
*V*_T_ (L)	0.60 ± 0.09	0.58 ± 0.13	0.552	1.14 ± 0.32	1.17 ± 0.26	0.492
$S_{pO_{2}}$ (%)	96 ± 2	97 ± 1	0.086	90 ± 5	92 ± 5	0.117
V˙E/V˙EV˙CO2V˙CO2	35.06 ± 4.09	34.27 ± 4.61	0.231	34.91 ± 5.22	34.49 ± 5.12	0.498
$P_{\mathrm{ET},CO_{2}}$(mmHg)	31.55 ± 2.82	32.32 ± 3.53	0.522	34.63 ± 4.16	35.66 ± 4.57	0.299
HR (beats/min)	85 ± 14	76 ± 11	0.002	124 ± 16	125 ± 19	0.918
HR reserve (%)	43 ± 10	48 ± 8	0.002	16 ± 11	16 ± 14	0.878
O_2_ pulse (mL/beat)	3.46 ± 0.66	3.65 ± 0.82	0.361	7.26 ± 1.57	7.35 ± 1.46	0.617
SBP (mmHg)	143 ± 22	144 ± 15	0.978	177 ± 27	182 ± 18	0.350
DBP (mmHg)	86 ± 10	88 ± 6	0.617	85 ± 13	85 ± 8	0.672

*Note*: *n* = 8 except *n* = 7 for SBP and DBP.

Abbreviations: DBP, diastolic blood pressure; *f*_R_, breathing frequency; HR, heart rate; $P_{\mathrm{ET},CO_{2}}$, end‐tidal partial pressure of carbon dioxide; RER, respiratory exchange ratio; SBP, systolic blood pressure; $S_{pO_{2}}$, oxygen saturation; ${\dot{V}}_{CO_{2}}$, carbon dioxide production; *V̇*_E_, minute ventilation; ${\dot{V}}_{O_{2}}$, oxygen consumption; *V*_T_, tidal volume.

### 3.3 Incremental CPET

**FIGURE 1:**
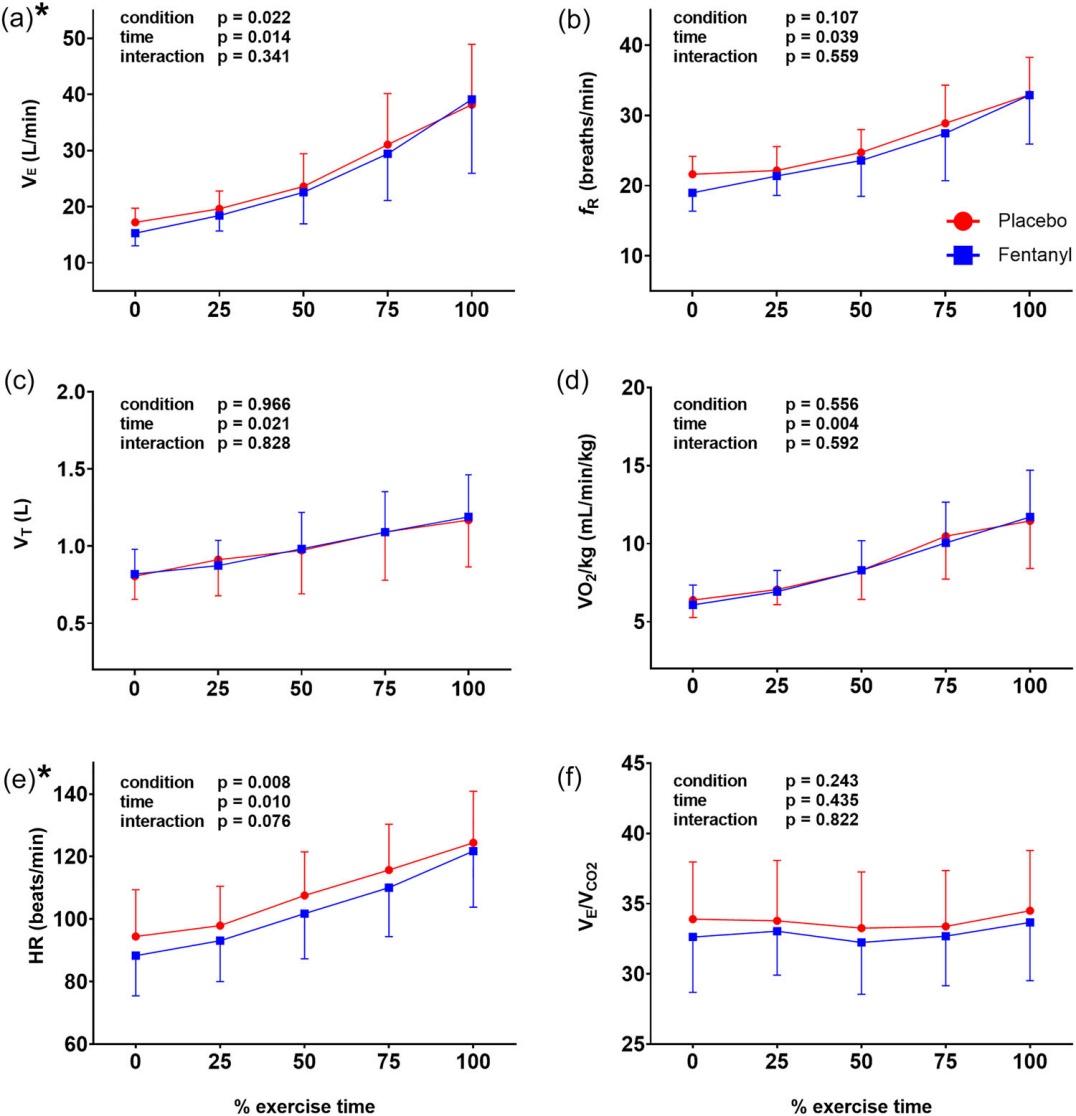
Minute ventilation (${\dot{V}}_{E}$; a), respiratory frequency (*f*_R_; b), tidal volume (*V*_T_; c), oxygen uptake (${\dot{V}}_{O_{2}}$/kg; d), heart rate (HR; e) and ventilatory equivalent for CO_2_ (V˙E/V˙EV˙CO2V˙CO2; f) during an incremental cardiopulmonary exercise test. *P < 0.05 fentanyl versus placebo.

**FIGURE 2:**
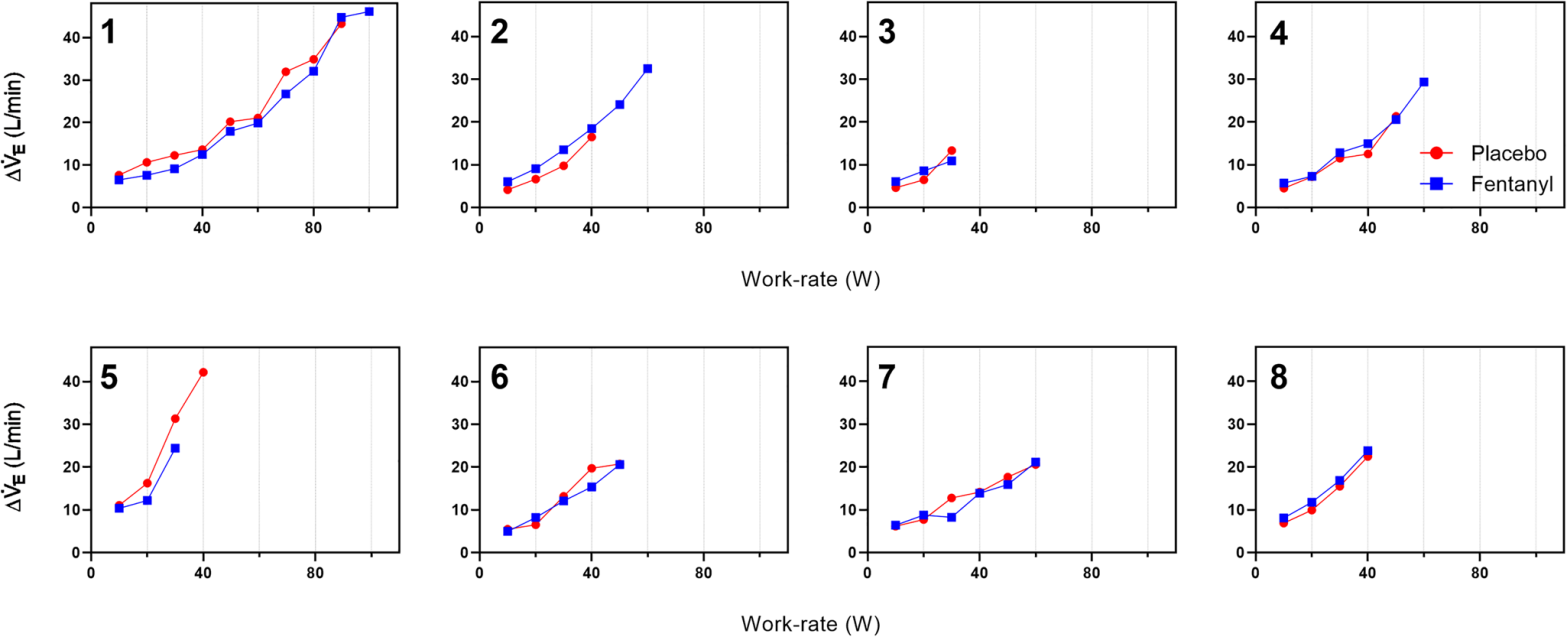
Change in minute ventilation (Δ*V̇*_E_) from baseline in individual participants (1–8).

Fentanyl did not change the EET (placebo 334 ± 117 s vs. fentanyl 348 ± 126 s, *P* = 0.250). Post‐fentanyl EET increased by >60 s in one participant only. Both conditions reached similar peak WR, and there were no differences between fentanyl and placebo at peak exercise (Table 1). Peak RER and HR reserve values indicated that the participants approached maximal effort during the CPET (American Thoracic Society & American College of Chest Physicians, 2003). During exercise, *V̇*_E_ was lower with fentanyl (Figure 1a) and *f*_R_ tended to be lower with fentanyl, although this did not reach statistical significance (*P* = 0.107). There was no difference in *V*_T_. Heart rate was also lower throughout the exercise after fentanyl compared with placebo (Figure 1e). There was no difference in the estimated lactate threshold between the conditions (placebo 8.49 ± 2.04 mL/min/kg vs. fentanyl 8.03 ± 1.82 mL/min/kg, *P* = 0.278). Individual Δ*V̇*_E_ compared with baseline during the last 15 s of each WR is displayed in Figure 2.

### 3.4 Symptoms

**FIGURE 3:**
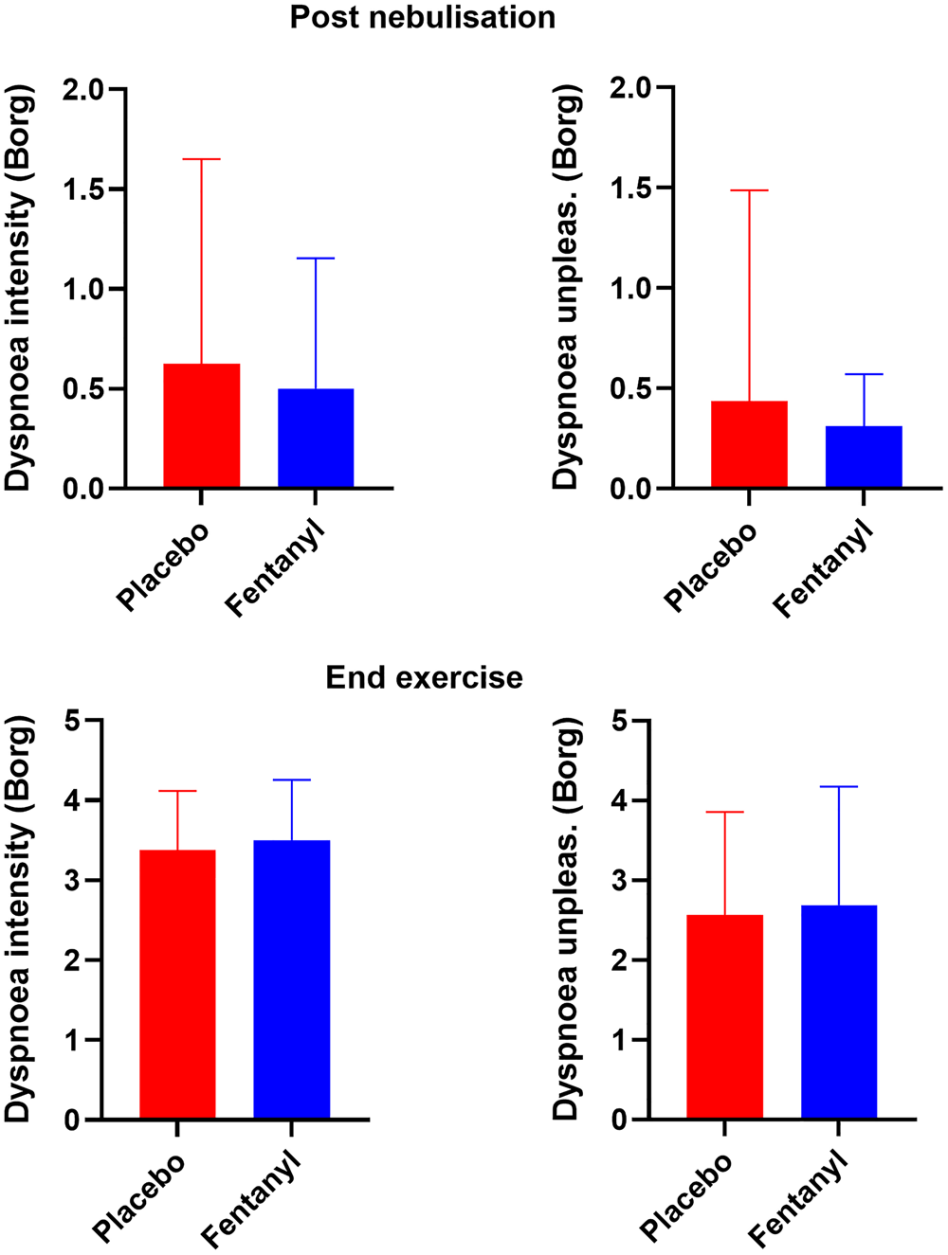
Ratings of the intensity and unpleasantness of dyspnoea post‐nebulization and at end exercise.

There was no overall difference in the intensity or unpleasantness of dyspnoea between placebo and fentanyl (Figure 3). Fentanyl did not change the rating of dyspnoea as a function of WR. Fentanyl did not change ratings of leg discomfort or RPE. In both conditions, the predominant reason for exercise termination was leg discomfort (*n* = 6 each). Blood fentanyl concentration was less than the lowest standard/negative quality control (<0.039 ng/mL) in all samples (unsuccessful phlebotomy *n* = 1). Six participants reported ‘yes’ to any symptom in the opioid‐related symptom distress scale in both fentanyl and placebo conditions. No participant required treatment with naloxone.

## 4 DISCUSSION

The aim of this study was to determine whether nebulized fentanyl could modify the cardiorespiratory response to whole‐body incremental exercise and lessen dyspnoea in patients with FILD. The major novel findings are as follows: (1) EET was not improved by fentanyl, although there was an overall reduction in *V̇*_E_ during exercise; (2) there was a reduction in HR post‐fentanyl at rest and throughout exercise; and (3) there was no improvement in the intensity or unpleasantness of dyspnoea after fentanyl compared with placebo. Although fentanyl caused a statistically significant (*P* = 0.022) reduction in overall *V̇*_E_, the difference was small in the context of relatively large SDs (Figure 1), and the individual data do not support a consistent effect of fentanyl on *V̇*_E_ (Figure 2). Furthermore, there was no difference in peak exercise *V̇*_E_ between fentanyl and placebo (Table 1). Therefore, we suggest that the reduction in exercise *V̇*_E_ observed is unlikely to be clinically significant.

Feedback mechanisms in the form of peripheral neural afferents constitute an important component in the control of the ventilatory response to exercise (Forster et al., 2011). The role of pulmonary vagal afferents is supported by studies of cycling exercise in lung and heart–lung transplant patients, where the vagus nerve was transected during surgery. Such patients demonstrated a reduction in the ventilatory response characterized by a more gradual increase in *f*_R_ (Sciurba et al., 1988), slower phase II response and inspiratory flow (Grassi et al., 1993). Evidence of opioids acting on pulmonary afferents has been shown both by direct application of morphine in human bronchi and indirectly, via right atrial injections in intact animals (Belvisi et al., 1992; Willette & Sapru, 1982). On this basis, nebulized fentanyl has been used as a strategy for attenuating pulmonary vagal afferents during exercise in patients with lung disease (Jensen et al., 2012; Milne et al., 2020). It is possible that the reduction in *V̇*_E_ observed with fentanyl in the present study is attributable to the modulation of pulmonary afferent feedback, although other mechanisms (e.g. systemic effects, statistical uncertainty) should be considered.

Aberrant pulmonary vagal activity could arise in inflammatory lung disease through the release of inflammatory mediators that either directly stimulate discharge or increase the sensitivity of the afferents (Mazzone & Undem, 2016). Both slowly adapting receptors and bronchopulmonary C‐fibres have been implicated in pulmonary inflammation or lung injury (Lin et al., 2007; Trenchard et al., 1972). The former mediates the Hering–Breuer inflation reflex, whereas activation of the latter causes rapid shallow breathing, bronchoconstriction and bradycardia (Lee & Yu, 2014). Neither reflex pathway accounts fully for the constellation of exercise *V̇_E_* and HR reduction observed herein. However, respiratory tract stimuli often affect multiple afferent pathways (Coleridge & Coleridge, 2011), resulting in a response that reflects a balance between inhibitory and excitatory effects (Lee & Yu, 2014). Owing to the lack of an age‐matched control group, our results do not provide direct evidence for the presence of aberrant signalling of pulmonary afferents in FILD. However, a previous study of nebulized fentanyl in healthy men found no effect on exercise *V̇*_E_ (Kotrach et al., 2015), hence this possibility cannot be excluded.

Our results contrast with those of Milne et al. (2020), who observed no differences in *V̇*_E_ between 100 µg fentanyl and placebo in persons with ILD. This discrepancy might be attributable to differences in the study design. Milne et al. (2020) used constant WR cycling, initially at 75% of peak WR, then titrated the WR between 60% and 90% of peak WR such that all participants performed 4 min of exercise. Although the exact WR used was not provided, we infer that the participants are likely to have performed tests of differing WR profiles between the two conditions. This limits the direct comparison of physiological response variables. Perhaps as a result, comparisons were made at only two time points, at rest and at 4 min. At 4 min, the ${\dot{V}}_{O_{2}}$ and HR of the participants had reached 92% of peak values of the incremental CPET (i.e., close to maximal exercise). There was a lack of data on responses during moderate‐intensity exercise, which is clinically relevant, because many ILD patients are likely to stop exercising much earlier than expected (Jones, 1997).

Dyspnoea is thought to be mediated through pulmonary vagal afferents, with second‐order neurons in the brainstem contributing to subcortical and cortical regions responsible for sensory processing and perception (Mazzone & Undem, 2016). In patients with cardiopulmonary disease, blockade of the cervical vagus nerve reduced dyspnoea (Guz et al., 1970), whereas stimulation of the vagus nerve either caused or intensified dyspnoea (Marks et al., 1996; Tetzlaff et al., 1999). The aforementioned link between inflammation and functional changes in vagal afferent neurons has been proposed as a mechanism for these observations (Mazzone & Undem, 2016). Inhaled fentanyl in COPD reduced the rate of increase in dyspnoea compared with placebo during cycle exercise (Jensen et al., 2012), which was not seen in our FILD participants. This discrepancy could reflect disease‐specific differences in pathology, with increased bronchomotor tone in COPD a target for nebulized fentanyl. Other reasons include exercise termination for non‐ventilatory reasons or submaximal exercise.

### 4.1 Experimental considerations

Multiple factors can contribute to the drive to breathe during exercise. It is acknowledged that in our study, inhaled fentanyl might have exerted effects via mechanisms beyond pulmonary vagal afferents. Respiratory depression induced by systemic opioids occurs by activation of µ‐opioid receptors in the brainstem respiratory network. However, this is unlikely to be the case in our participants, because the change in *V̇*_E_ was exercise specific and blood fentanyl concentrations were not increased.

This study has several limitations in addition to those already discussed. Our sample size was relatively small, and we did not carry out tests of within‐subject reproducibility nor validation that maximal ${\dot{V}}_{O_{2}}$ had been achieved. Both nebulized fentanyl and CPETs are not risk‐free interventions, even more so with FILD participants. Therefore, we had an ethical obligation to balance study power and unnecessary participant risk. A priori sample size calculation was based on nebulized fentanyl in COPD data (Jensen et al., 2012), because at the time the work of Milne et al. (2020) had not been published. To detect differences in Borg dyspnoea ratings of ±1.5, EET of 86 s and peak *V̇*_E_ of 0.5 L/min, it was estimated that 15 patients would be required (power = 0.8, 5% α). We acknowledge that because the target sample size was not reached, our study will have reduced statistical power. However, after eight participants there was no clear evidence of a difference in the primary outcomes of EET and dyspnoea intensity. In addition, we felt that taking into account the study by Milne et al. (2020), a similar but larger study with negative findings, it was more difficult to justify ongoing recruitment in terms of participant welfare and safety, particularly given that our patients probably had more severe disease (mean forced vital capacity 65% vs. 78% of predicted). We acknowledge that this limitation is likely to be compounded by the choice of using an incremental WR test, which has lower sensitivity for changes in post‐interventional EET (Casaburi, 2005). However, the use of EET in constant WR tests is not without its own challenges, owing to the power–duration relationship (Whipp & Ward, 2009). Finally, our study does not provide direct evidence of the effect of fentanyl on pulmonary afferents, because recording of these afferents is not possible in intact humans.

## 5 CONCLUSION

Nebulized fentanyl citrate (100 µg) reduced *V̇*_E_ during exercise in FILD; however, the extent of this effect is unlikely to be clinically significant and there was no improvement in EET. A systemic opioid effect appears unlikely at this dose. On the basis of the present study and previously published data, there is no evidence to support the hypothesis that nebulized fentanyl can lead to significant improvements in exercise tolerance or exertional dyspnoea.

## AUTHOR CONTRIBUTIONS

Conception and design of the work: Charlotte Chen, John Kolbe, Julian F. R. Paton and James P. Fisher. Data acquisition: Charlotte Chen. Analysis: Charlotte Chen. Interpretation of data: Charlotte Chen, Julian F. R. Paton and James P. Fisher. Drafting of the work: Charlotte Chen. All authors reviewed the work critically for important intellectual content, gave approval for the version to be published and agreed to be accountable for all aspects of the work in ensuring that questions related to the accuracy or integrity of any part of the work are appropriately investigated and resolved. All persons designated as authors qualify for authorship, and all those who qualify for authorship are listed. Experiments were performed in the Human Cardiorespiratory Physiology Laboratory, Auckland City Hospital, Te Whatu Ora – Health New Zealand Te Toka Tumai Auckland.

## CONFLICT OF INTEREST

None declared.

## Supporting information



S1. Full list of exclusion criteria.
S2. Medical conditions (excluding FILD‐related conditions) of participants.
S3. Long‐term prescription medication of participants.
S4. Breathing pattern and variability at rest.
S5. Post‐nebulization spirometry.

## Data Availability

Data are available upon reasonable request to the corresponding author.

## References

[eph13670-bib-0001] American Thoracic Society & American College of Chest Physicians . (2003). An official American Thoracic Society statement: ATS/ACCP Statement on cardiopulmonary exercise testing. American Journal of Respiratory and Critical Care Medicine, 167(2), 211–277. 12524257 10.1164/rccm.167.2.211

[eph13670-bib-0002] Apfelbaum, J. L. , Gan, T. J. , Zhao, S. , Hanna, D. B. , & Chen, C. (2004). Reliability and validity of the perioperative opioid‐related symptom distress scale. Anesthesia & Analgesia, 99(3), 699–709. 15333398 10.1213/01.ANE.0000133143.60584.38

[eph13670-bib-0003] Bajwah, S. , Ross, J. R. , Peacock, J. L. , Higginson, I. J. , Wells, A. U. , Patel, A. S. , Koffman, J. , & Riley, J. (2013). Interventions to improve symptoms and quality of life of patients with fibrotic interstitial lung disease: A systematic review of the literature. Thorax, 68(9), 867–879. 23204065 10.1136/thoraxjnl-2012-202040

[eph13670-bib-0004] Barnes, H. , McDonald, J. , Smallwood, N. , & Manser, R. (2016). Opioids for the palliation of refractory breathlessness in adults with advanced disease and terminal illness. Cochrane Database of Systematic Reviews, 3(3), CD011008. 27030166 10.1002/14651858.CD011008.pub2 PMC6485401

[eph13670-bib-0005] Beaver, W. L. , Wasserman, K. , & Whipp, B. J. (1986). A new method for detecting anaerobic threshold by gas exchange. Journal of Applied Physiology (1985), 60(6), 2020–2027. 10.1152/jappl.1986.60.6.2020 3087938

[eph13670-bib-0006] Belvisi, M. G. , Stretton, C. D. , Verleden, G. M. , Ledingham, S. J. , Yacoub, M. H. , & Barnes, P. J. (1992). Inhibition of cholinergic neurotransmission in human airways by opioids. Journal of Applied Physiology (1985), 72(3), 1096–1100. 10.1152/jappl.1992.72.3.1096 1314798

[eph13670-bib-0007] Borg, G. A. (1982). Psychophysical bases of perceived exertion. Medicine and Science in Sports and Exercise, 14(5), 377–381. 7154893

[eph13670-bib-0008] Broaddus, C. , Mason, R. , Ernst, J. , King, T. , Lazarus, S. , Murray, J. , Nadel, J. , Slutsky, A. , & Gotway, M. (2016). Murray & Nadel's textbook of respiratory medicine (6th ed.). Elsevier Saunders.

[eph13670-bib-0009] Casaburi, R. (2005). Factors determining constant work rate exercise tolerance in COPD and their role in dictating the minimal clinically important difference in response to interventions. Chronic Obstructive Pulmonary Disease, 2(1), 131–136. 10.1081/copd-200050576 17136973

[eph13670-bib-0010] Coleridge, H. , & Coleridge, J. (2011). Reflexes evoked from tracheobronchial tree and lungs. In Comprehensive *p*hysiology (pp. 395–429). John Wiley & Sons.

[eph13670-bib-0011] Cottin, V. , Wollin, L. , Fischer, A. , Quaresma, M. , Stowasser, S. , & Harari, S. (2019). Fibrosing interstitial lung diseases: Knowns and unknowns. European Respiratory Review, 28(151), 180100. 30814139 10.1183/16000617.0100-2018 PMC9489101

[eph13670-bib-0012] Coyne, P. J. , Viswanathan, R. , & Smith, T. J. (2002). Nebulized fentanyl citrate improves patients' perception of breathing, respiratory rate, and oxygen saturation in dyspnea. Journal of Pain and Symptom Management, 23(2), 157–160. 11844637 10.1016/s0885-3924(01)00391-8

[eph13670-bib-0013] Forster, H. V. , Haouzi, P. , & Dempsey, J. A. (2011). Control of breathing during exercise. Comprehensive Physiology, 2(1), 743–777. 10.1002/cphy.c100045 23728984

[eph13670-bib-0014] Grassi, B. , Ferretti, G. , Xi, L. , Rieu, M. , Meyer, M. , Marconi, C. , & Cerretelli, P. (1993). Ventilatory response to exercise after heart and lung denervation in humans. Respiration Physiology, 92(3), 289–304. 8351447 10.1016/0034-5687(93)90014-2

[eph13670-bib-0015] Guz, A. , Noble, M. , Eiseled, H. , & Trenchard, D. (1970). Experimental results of vagal block in cardio‐pulmonary disease. In Breathing: Hering‐Breuer centenary symposium (pp. 315–336). Churchill.

[eph13670-bib-0016] Jensen, D. , Alsuhail, A. , Viola, R. , Dudgeon, D. J. , Webb, K. A. , & O'Donnell, D. E (2012). Inhaled fentanyl citrate improves exercise endurance during high‐intensity constant work rate cycle exercise in chronic obstructive pulmonary disease. Journal of Pain and Symptom Management, 43(4), 706–719. 22168961 10.1016/j.jpainsymman.2011.05.007

[eph13670-bib-0017] Jones, N. L. (1997). Clinical exercise testing (4th ed.). Saunders.

[eph13670-bib-0018] Keir, D. A. , Iannetta, D. , Mattioni Maturana, F. , Kowalchuk, J. M. , & Murias, J. M. (2022). Identification of non‐invasive exercise thresholds: Methods, strategies, and an online app. Sports Medicine, 52(2), 237–255. 34694596 10.1007/s40279-021-01581-z

[eph13670-bib-0019] Kotrach, H. G. , Bourbeau, J. , & Jensen, D. (2015). Does nebulized fentanyl relieve dyspnea during exercise in healthy man? Journal of Applied Physiology (1985), 118(11), 1406–1414. 10.1152/japplphysiol.01091.2014 PMC4451291 26031762

[eph13670-bib-0020] Krajnik, M. , Schäfer, M. , Sobański, P. , Kowalewski, J. , Bloch‐Bogusławska, E. , Zylicz, Z. , & Mousa, S. A. (2010). Local pulmonary opioid network in patients with lung cancer: A putative modulator of respiratory function. Pharmacological Reports, 62(1), 139–149. 20360624 10.1016/s1734-1140(10)70251-6

[eph13670-bib-0021] Lee, L. Y. , & Yu, J. (2014). Sensory nerves in lung and airways. Comprehensive Physiology, 4(1), 287–324. 24692141 10.1002/cphy.c130020

[eph13670-bib-0022] Lin, S. , Walker, J. , Xu, L. , Gozal, D. , & Yu, J. (2007). Behaviours of pulmonary sensory receptors during development of acute lung injury in the rabbit. Experimental Physiology, 92(4), 749–755. 17392336 10.1113/expphysiol.2006.036673

[eph13670-bib-0023] Manali, E. D. , Stathopoulos, G. T. , Kollintza, A. , Kalomenidis, I. , Emili, J. M. , Sotiropoulou, C. , Daniil, Z. , Roussos, C. , & Papiris, S. A. (2008). The Medical Research Council chronic dyspnea score predicts the survival of patients with idiopathic pulmonary fibrosis. Respiratory Medicine, 102(4), 586–592. 18162388 10.1016/j.rmed.2007.11.008

[eph13670-bib-0024] Marks, G. , Yates, D. , Sist, M. , Ceyhan, B. , De Campos, M. , Scott, D. , & Barnes, P. (1996). Respiratory sensation during bronchial challenge testing with methacholine, sodium metabisulphite, and adenosine monophosphate. Thorax, 51(8), 793–798. 8795666 10.1136/thx.51.8.793 PMC472540

[eph13670-bib-0025] Mazzone, S. B. , & Undem, B. J. (2016). Vagal afferent innervation of the airways in health and disease. Physiological Reviews, 96(3), 975–1024. 27279650 10.1152/physrev.00039.2015 PMC4982036

[eph13670-bib-0026] Milne, K. M. , Ibrahim‐Masthan, M. , Scheeren, R. E. , James, M. D. , Phillips, D. B. , Moran‐Mendoza, O. , Neder, J. A. , & O'Donnell, D. E. (2020). Inspiratory neural drive and dyspnea in interstitial lung disease: Effect of inhaled fentanyl. Respiratory Physiology & Neurobiology, 282, 103511. 32758677 10.1016/j.resp.2020.103511

[eph13670-bib-0027] Noble, M. , Eiseled, H. , Trenchard, D. , & Guz, A. (1970). Effect of selective peripheral nerve blocks on respiratory sensations. In Ciba Foundation symposium‐breathing: Hering‐Breuer centenary symposium (pp. 233–251). Wiley Online Library.

[eph13670-bib-0028] Sciurba, F. C. , Owens, G. R. , Sanders, M. H. , Griffith, B. P. , Hardesty, R. L. , Paradis, I. L. , & Costantino, J. P. (1988). Evidence of an altered pattern of breathing during exercise in recipients of heart–lung transplants. New England Journal of Medicine, 319(18), 1186–1192. 3140013 10.1056/NEJM198811033191803

[eph13670-bib-0029] Sverzellati, N. , Lynch, D. A. , Hansell, D. M. , Johkoh, T. , King Jr., T. E. , , & Travis, W. D (2015). American Thoracic Society–European Respiratory Society classification of the idiopathic interstitial pneumonias: Advances in knowledge since 2002. Radiographics, 35(7), 1849–1871. 26452110 10.1148/rg.2015140334

[eph13670-bib-0030] Swigris, J. J. , Kuschner, W. G. , Jacobs, S. S. , Wilson, S. R. , & Gould, M. K. (2005). Health‐related quality of life in patients with idiopathic pulmonary fibrosis: A systematic review. Thorax, 60(7), 588–594. 15994268 10.1136/thx.2004.035220 PMC1747452

[eph13670-bib-0031] Tetzlaff, K. , Leplow, B. , ten Thoren, C. , & Dahme, B. (1999). Perception of dyspnea during histamine‐and methacholine‐induced bronchoconstriction. Respiration, 66(5), 427–433. 10516539 10.1159/000029426

[eph13670-bib-0032] Trenchard, D. , Gardner, D. , & Guz, A. (1972). Role of pulmonary vagal afferent nerve fibres in the development of rapid shallow breathing in lung inflammation. Clinical Science, 42(3), 251–263. 5013871 10.1042/cs0420251

[eph13670-bib-0033] Whipp, B. J. , & Ward, S. A. (2009). Quantifying intervention‐related improvements in exercise tolerance. European Respiratory Journal, 33(6), 1254–1260. 19483045 10.1183/09031936.00110108

[eph13670-bib-0034] Willette, R. N. , & Sapru, H. N. (1982). Pulmonary opiate receptor activation evokes a cardiorespiratory reflex. European Journal of Pharmacology, 78(1), 61–70. 6281030 10.1016/0014-2999(82)90372-7

[eph13670-bib-0035] Worsley, M. H. , MacLeod, A. D. , Brodie, M. J. , Asbury, A. J. , & Clark, C. (1990). Inhaled fentanyl as a method of analgesia. Anaesthesia, 45(6), 449–451. 2382801 10.1111/j.1365-2044.1990.tb14331.x

[eph13670-bib-0036] Zebraski, S. E. , Kochenash, S. M. , & Raffa, R. B. (2000). Lung opioid receptors: Pharmacology and possible target for nebulized morphine in dyspnea. Life Sciences, 66(23), 2221–2231. 10855942 10.1016/s0024-3205(00)00434-3

