# Supplementary material for: Nebulized fentanyl does not improve exercise capacity or dyspnoea in fibrosing interstitial lung disease
Source: Exp Physiol. 2024 Oct 12;110(1):15–22. doi: 10.1113/EP092123 (PMC11689134; doi:10.1113/EP092123)
Supplement: Supplementary file 1 — S1. Full list of exclusion criteria. S2. Medical conditions (excluding FILD‐related conditions) of participants. S3. Long‐term prescription medication of participants. S4. Breathing pattern and variability at rest. S5. Post‐nebulization spirometry. [file EPH-110-15-s001.docx]

Supplementary

|  |  |
| --- | --- |

S1. Full list of exclusion criteria

- More than 15 pack year smoking history
- Evidence of emphysema on CT scan
- Pulmonary Sarcoidosis
- Currently taking regular inhaled therapy for airways disease
- Serious co-morbidities that may contribute to dyspnoea and/or reduce exercise capacity including:
  - Severe respiratory disease other than ILD (e.g., chronic obstructive pulmonary disease)
  - Pulmonary hypertension
  - Severe obesity (Body mass index > 35 kg/m^2^)
  - Severe orthopaedic impairment or rheumatologic disease
  - Significant neurological disease
  - Infection or pyrexial illness
- Presence of any contraindications to cardiopulmonary exercise testing
  - Unstable angina or recent acute myocardial infarction
  - Uncontrolled arrhythmias causing symptoms or haemodynamic compromise
  - Symptomatic severe aortic stenosis
  - Oxygen saturation <85% at rest on room air
  - Uncontrolled heart failure
  - Uncontrolled asthma
  - Uncontrolled thyroid disorders
  - Mental impairment leading to inability to cooperate
- Current pregnancy
- Allergy or intolerance to fentanyl
- Use of anti-depressants (ie monoamine oxidase inhibitors, serotonin reuptake inhibitors, serotonin norephinephrine re-uptake inhibitors) in the last 14 days.
- Use of opioid medications (eg morphine, fentanyl, oxycodone, hydromorphone, methadone, codeine) in the previous 4 weeks.
- Current users of recreational drugs
- Current abusers of alcohol
- Inability to fully or appropriately provide consent (e.g., language issue, reading capability)
- Underlying medical conditions, which in the opinion of the Investigator place the participant at unacceptably high risk for participating in the study.

|  |
| --- |

S2. Medical conditions (excluding FILD-related conditions) of participants

| Condition type | Number of patients |
| --- | --- |
| Hypertension | 3 |
| Previous cancer | 2 |
| Dyslipidemia | 3 |
| Gastroesophageal reflux disease | 3 |
| Allergic rhinitis | 2 |
| Osteoporosis | 1 |
| Hypothyroidism | 1 |
| Monoclonal gammopathy | 1 |

|  |  |
| --- | --- |

S3. Long-term prescription medication of participants

| Medication type | Number of patients |
| --- | --- |
| Non-corticosteroid immunosuppressant | 3 |
| Corticosteroid | 4 |
| Anti-hypertensive | 3 |
| Anti-reflux | 4 |
| Statin | 2 |
| Bone-protection agents | 5 |
| Anti-fibrotic | 2 |
| Hormone replacement | 2 |

S4. Breathing pattern and variability at rest

|  | Raw data | | | Coefficient of variability | | |
| --- | --- | --- | --- | --- | --- | --- |
|  | Placebo | Fentanyl | *p* value | Placebo | Fentanyl | *p* value |
| Inspiratory time (s) | 1.37 ± 0.11 | 1.41 ± 0.20 | 0.600 | 0.24 ± 0.08 | 0.20 ± 0.09 | 0.089 |
| Expiratory time (s) | 1.86 ± 0.28 | 1.97 ± 0.51 | 0.400 | 0.26 ± 0.08 | 0.22 ± 0.07 | 0.201 |
| Inspiratory time / total time (%) | 43 ± 3 | 42 ± 4 | 0.782 | 15 ± 4 | 13 ± 5 | 0.321 |
| Tidal volume (L) | 0.60 ± 0.09 | 0.58 ± 0.14 | 0.480 | 0.23 ± 0.10 | 0.21 ± 0.06 | 0.532 |

S5. Post-nebulisation spirometry

|  | Placebo | Fentanyl | *p* value |
| --- | --- | --- | --- |
| FEV_1_ (L) | 1.70 ± 0.51 | 1.65 ± 0.49 | 0.440 |
| FEV% pred. (%) | 70.00 ± 16.80 | 68.00 ± 16.95 | 0.449 |
| FVC (L) | 2.01 ± 0.69 | 1.96 ± 0.63 | 0.446 |
| FVC% pred. (%) | 63.25 ± 16.05 | 61.63 ± 14.71 | 0.392 |
| FEV_1_/FVC | 86 ± 6 | 85 ± 10 | 0.796 |

FEV_1_, forced expiratory volume in one second; FVC, forced vital capacity.
